# Cataract Surgery in Very Old Patients: A Case-Control Study

**DOI:** 10.3390/jcm10204658

**Published:** 2021-10-11

**Authors:** Hanan Nussinovitch, Erez Tsumi, Raimo Tuuminen, Boris Malyugin, Yotam Lior, Hadar Naidorf Rosenblatt, Matthew Boyko, Asaf Achiron, Boris Knyazer

**Affiliations:** 1 Department of Ophthalmology, Shaarei Zedek Medical Center, Jerusalem 9103102, Israel; hananinio@gmail.com; 2 Department of Ophthalmology, Soroka University Medical Center, Faculty of Health Sciences, Ben-Gurion University of the Negev, Beersheba 8410501, Israel; ertsumi@bgu.ac.il; 3 Helsinki Retina Research Group, Faculty of Medicine, University of Helsinki, 00100 Helsinki, Finland; raimo.tuuminen@helsinki.fi; 4 Department of Ophthalmology, Kymenlaakso Central Hospital, 48210 Kotka, Finland; 5 S. Fyodorov Eye Microsurgery Federal State Institution, 127486 Moscow, Russia; boris.malyugin@gmail.com; 6 Department of Eye Diseases, A.I. Yevdokimov Moscow State University of Medicine and Dentistry, 127473 Moscow, Russia; 7 Division of Anesthesiology and Critical Care, Soroka University Medical Center and the Faculty of Health Sciences, Ben-Gurion University of the Negev, Beersheba 8410501, Israel; matoyster@gmail.com (Y.L.); matthewboykoresearch@gmail.com (M.B.); 8 Ophthalmology Department, Kaplan Medical Center, Rehovot 7610001, Israel; hadarnaidorf@gmail.com; 9 Tel Aviv Sourasky Medical Center, Sackler Faculty of Medicine, Tel Aviv University, Tel-Aviv 6997801, Israel

**Keywords:** cataract surgery, very old patients, visual acuity gain

## Abstract

Advancements in surgical techniques and increased life expectancy have made cataract surgery more common among very old patients. However, surgical outcomes seem impaired in patients older than 90 years, especially with ocular comorbidities. A retrospective case-control study of 53 eyes of 53 very old patients (mean 92.6 ± 3.0) and 140 eyes of 140 matched patients (mean 75.2 ± 7.6) was undertaken. Groups were matched in terms of gender and systemic and ocular comorbidities. In very old patients, higher phacoemulsification energy (cumulative dissipated energy [CDE], 25.0 ± 22.4 vs. 16.1 ± 10.7, *p* = 0.01) and rate of intraoperative floppy iris syndrome (IFIS, 9.4% vs. 1.4%, *p* = 0.02) were observed compared to controls. Uncorrected (UCVA) and best-corrected distance visual acuity (BCVA) gains were significantly poorer among the very old patients than among the control at postoperative day 30 (0.20 ± 0.70 vs. 0.56 ± 0.61 logMAR, *p* < 0.001 and 0.27 ± 0.64 vs. 0.55 ± 0.62 logMAR, *p* = 0.006, respectively). Even after including CDE and IFIS as covariates, age remained an independent factor for poor visual gain at 30 days (*p* < 0.001). Cataract surgery in very old patients may demand more experienced surgeons due to higher nuclear density and the rates of IFIS. Expectations in visual acuity gains should be aligned with the patient’s age.

## 1. Introduction

Cataracts are the most common cause of reversible visual impairment in the developed world. Nearly half of people in their 70s and almost all in their 90s suffer from significant cataracts [1,2]. Furthermore, the steady rise in lifespan worldwide has been accompanied by an increased need for cataract surgical intervention at extreme ages [3].

Studies on cataract surgery outcomes in the elderly population (>85 or >90 years) have been reported in Japan [1], Sweden [2,4], UK [5,6,7], Denmark [8], Germany [9], Korea [10], USA [11,12,13], China [14], Turkey [15], and Israel [16]. However, none of these studies included a matched control in systemic and ocular comorbidity, which may confound the age effect [17]. 

Here, we aimed to analyze visual acuity gain after cataract surgery in the elderly population (≥90 years) compared to demographics, and systemic and ocular comorbidity matched younger patients.

## 2. Materials and Methods

### 2.1. Study Design

This was a consecutive case series, and we reviewed all patients who underwent cataract surgery from September 2009 to August 2016 at the Department of Ophthalmology, Soroka University Medical Central, a tertiary hospital in Beersheba, southern Israel. Exclusion criteria included corneal diseases, glaucoma, wet age-related macular degeneration (wAMD), diabetic retinopathy (DR), prior history of retinal detachment and retinal vascular diseases, and follow-up cases less than one month.

Data retrieved from the hospital’s electronic database included age, sex, ocular and systemic comorbidity, intraoperative complications, cumulative dissipated energy (CDE), pre-and postoperative uncorrected distance visual acuity (UCVA), best-corrected distance visual acuity (BCVA), and ocular slit-lamp examinations of the cataract types.

The local Institutional Review Board approved the study of the Ethics Committee at Soroka University Medical center, and the design and data collection complied with all local laws and the Declaration of Helsinki principles. 

### 2.2. Cataract Surgery and Follow-Up Data

Cataract surgery was performed in all patients through a clear corneal incision under topical anesthesia using an Infiniti^®^ phacoemulsification unit (Alcon, Fort Worth, TX, USA). Intraoperative complications were recorded in all patients. Patients were postoperatively evaluated at seven and 30 days. Patients with a normal postoperative clinical course moved to community-based ophthalmology clinics for follow-up. Those with complications or unsatisfactory post-surgical results remained in the department for follow-up. Data up to one month of follow-up was collected from patients who remained under observation in the department. Only senior surgeons and young consultants (>4 years of surgery experience with over 150 cases) had operated on the older subjects group, as our department’s protocol considers an age over 88 years to be a significant risk factor [18].

### 2.3. Statistical Analysis

Statistical analyses were performed using IBM SPSS Statistics 27 (SPSS Inc., Chicago, IL, USA). Snellen values were converted to logarithm of the minimum angle of resolution (logMAR) for statistical purposes. In visual acuity testing, when the largest optotype could not be recognized correctly, the classification was very low visual acuity (VA) on a semi-quantitative scale such as counting fingers (CF), hand motion (HM), and light perception (LP). The very low VA measurements have been converted as follows: CF to 2, HM to 3, and LP to 4 logarithm of the minimum angle of resolution (logMAR) units. Parametric model assumptions were assessed using Normal-plot or Shapiro-Wilks test to verify normality and Levene’s test to verify the homogeneity of variances. A parametric Student’s t-test analyzed continuous variables. When parametric assumptions were not satisfactory even after data transformation attempts, the Mann-Whitney nonparametric test was used. Categorical variables were tested using Pearson’s χ2 test for contingency tables or the Fisher Exact test. Logistic regression models were constructed and presented with 95% CI and OR where considered appropriate. ANCOVA was used to remove the effect of the covariate factors, including the presence of brunescent cataracts and the use of α-antagonists or the level of phacoemulsification energy and presence of intraoperative floppy iris syndrome (IFIS). All statistical tests and confidence intervals were performed at α = 0.05 (2-sided).

## 3. Results

### 3.1. Baseline Parameters

Of the 12,663 patients who underwent cataract surgery from September 2009 to August 2016, 160 patients were 90 years of age or older (1.2%). Based on our exclusion criteria, the study cohort consisted of 53 eyes of 53 patients ≥90 years of age at the time of surgery (“older group”, mean age 92.6 ± 3.0 years) and 140 eyes of 140 patients of 50–89 years of age (“younger group”, mean age 75.2 ± 7.6 years). These two groups were matched for systemic comorbidity as DM, hypertension, dyslipidemia, stroke history, and obesity (Table 1). 

It is worth noting that the patients were not matched in terms of cataract severity and risk for IFIS: the very old patients had a higher rate of oral α-antagonist treatment (13.2% vs. 2.9%, *p* = 0.01, Table 1) and IFIS (9.4% vs. 1.4%, *p* = 0.02, Table 2), as well as a higher rate of brunescent cataract (17.0% vs. 6.4%, *p* = 0.02, Table 1) and the level of phacoemulsification energy (24.9 ± 22.4 CDE vs. 16.1 ± 10.7 CDE, *p* = 0.01, Table 2) when compared to the control cohort. There were no group differences in terms of intraoperative use of pupil-expanding devices (*p* = 0.4) or capsular tension rings (*p* = 0.71), nor in rates of posterior capsule rupture (*p* = 0.33) or anterior vitrectomy (*p* = 0.11).

### 3.2. Visual Acuity Gains after Cataract Surgery

At seven days, postoperative UCVA and BCVA gain were poorer in the very old patients (−0.02 ± 0.66 vs. 0.35 ± 0.69, *p* < 0.001 and 0.04 ± 0.73 vs. 0.39 ± 0.65, *p* = 0.001, respectively, Table 3) when compared to the controls. 

At 30 days, postoperative UCVA and BCVA gain were poorer in the very old patients (0.20 ± 0.70 vs. 0.56 ± 0.61, *p* < 0.001 and 0.27 ± 0.64 vs. 0.55 ± 0.62, *p* = 0.006, respectively, Table 3) when compared to the controls.

Rate of UCVA gain of at least three lines was significantly lower in the very old patients at seven days (24.5% vs. 42.9%, *p* = 0.019, Table 4) and at 30 days (26.4% vs. 53.6%, *p* = 0.001, Table 4) when compared to the younger group. Rate of BCVA gain of at least three lines was significantly lower in very old patients at 30 days (30.2% vs. 52.1%, *p* = 0.006, Table 4) compared to the control cohort. 

Patient age at surgery, both as a linear parameter and as a binary variable (very old patients vs. cohort), remained an independent factor for poor visual gain at 30 days even after significant pre-and intraoperative covariants; either the presence of brunescent cataract and the use of α-antagonists, or the level of phacoemulsification energy and the presence of IFIS were included in the analysis (Table 5). When the presence of brunescent cataract and the use of α-antagonists were included as covariants, in the matched control group UCVA and BCVA gains at 30 days were 0.393 (95% CI 0.182-0.605) LogMAR units higher, *p* < 0.001 and 0.317 (95% CI 0.108–0.526) LogMAR units higher, *p* = 0.003, respectively, compared to the very old patients. When phacoemulsification energy and IFIS were included as covariants, in the matched control, UCVA and BCVA gains at 30 days were 0.430 (95% CI 0.212-0.647) LogMAR units higher, *p* < 0.001 and 0.345 (95% CI 0.129–0.562) LogMAR units higher, *p* = 0.002, respectively, compared to the very old patients. 

## 4. Discussion

There is conflicting evidence as to whether older age is a significant predictor of worse visual acuity outcomes. Eight of the 17 reviewed studies had included a control group, but none had matched ocular and systemic comorbidity (Supplementary Table S1). To analyze patient age at cataract surgery as an independent parameter, we had set a case-control design with an aim to reduce confounding variables as much as possible.

We found that in very old patients, the incidence of brunescent cataracts was higher when compared to the control group, which resulted in higher phacoemulsification energy during surgery. While the study groups were matched for ocular and systemic comorbidities, the very old patients more frequently used α-antagonists and had a higher occurrence of IFIS. In patients over 90 years, Rosen et al. reported visual acuity improvement in only 68% of patients [16]. In contrast, Mönestam and Wachmeister found that visual acuity improved after cataract surgery in 94% of patients [2]. Visual acuity improvement in very old patients translated to improved quality-of-life, postural stability, and reduction of fall-related fractures [19]. We did not find any differences in the rate of posterior capsule rupture (PCR) between the very old patients and the controls, which is in accordance with the studies by Mutoh et al. [1] and Robbie et al. [20], who found no significant differences in the rate of intraoperative complications of cataract surgery in patients aged above or below 90 years. Furthermore, similar to previous literature, we did not find any differences in the use of pupil expansion devices between the two groups. In other studies, however, advanced age (not necessary >90 years) was found to be correlated witha posterior capsule rupture, raised intraocular pressure, endophthalmitis, and corneal edema [20,21].

The higher incidence of brunescent cataracts in very old patients can be explained by the correlation between age and increased exposure to sunlight in our subtropical area [17]. Zigman et al. [18] noted that chronic exposure to sunlight explicitly enhances lenticular transformation and brunescent cataract development due to human lens insoluble protein aggregations consisting of increasing concentrations of the photo-oxidized form of the amino acid tryptophan. This form of tryptophan increases lens cell toxicity, pigmentation, and the formation of lens protein aggregates, which lead to decreased light transmittance and brunescent cataract formation [19]. Advanced cataracts in the elderly group resulted in significantly higher phacoemulsification energy. This may lead to ultrasound-induced corneal endothelial cell damage with subsequent corneal edema as a possible mechanism for this group’s reduced early postoperative visual acuity [20]. 

Analysis including pre-and intraoperative covariants showed that patient age at cataract surgery, both as a linear parameter and as a group (very old patients vs. controls), remained an independent factor for poor visual gain at 30 days. Mutoh et al. suggested that poorer postoperative results in patients over 90 years were due to preoperative systemic disease and intraoperative changes in systemic conditions of the patients at this age [1]. Berler reviewed 102 eyes of patients aged 88–98 years in whom no highly dense cataracts were found [19]. He concluded that age and comorbidities, rather than more advanced cataracts, explained the increased rate of intraoperative complications and worse postoperative VA in the elderly [19]. 

The study limitations are its retrospective design and limited postoperative follow-up with missing data for endothelial cell counts, central corneal thickness, and macular thickness. This hinders the estimation of post-surgery posterior capsule opacification, macular edema, bullous keratopathy, surgery-induced astigmatism, and other rarer complications (such as IOL subluxation or retinal detachment). In addition, surgeries were performed by several surgeons with different levels of expertise that might have increased the variability of postoperative results. On the other hand, having surgeons with variable levels of expertise reflects real-life clinical practice. Finally, the relatively short VA follow-up also limits the estimation of results in the long term.

To conclude, VA gains were lower in the very old patients when compared to the matched cohort of younger patients. Our findings can help the cataract surgeons, patients, and their caregivers estimate cataract surgery outcomes on visual acuity and quality of life for this population. The higher rate of preoperative oral α-antagonist treatment and the more advanced cataracts in the very old patients highlights the importance of preoperative counseling. This will benefit the elderly candidates and their caregivers regarding goals and expectations and may reduce complications by referring them to a more experienced surgeon.

## Figures and Tables

**Table 1:** Intraoperative procedures and complications in study populations.

Variable	Age 50–89 Years *n* = 132	Age ≥90 Years *n* = 53	*p*-Value
Phacoemulsification energy (CDE)	16.1 ± 10.7	24.9 ± 22.4	0.01
IFIS	2 (1.4)	5 (9.4)	0.02
Pupil expanding device	29 (20.7)	14 (26.4)	0.40
CTR	6 (4.3)	3 (5.7)	0.71
Posterior capsule rupture	5 (3.6)	0 (-)	0.33

Data is given as mean (±SD) or absolute numbers and proportions. CDE; cumulative dissipated energy, CTR; capsular tension ring, IFIS; intraoperative floppy iris syndrome, IOL; intraocular lens.

**Table 2:** Intraoperative procedures and complications in study populations.

Variable	Age 50–89 Years *n* = 140	Age ≥90 Years *n* = 53	*p*-Value
Phacoenergy (CDE)	16.1 ± 10.7	24.9 ± 22.4	0.01
IFIS	2 (1.4)	5 (9.4)	0.02
Pupil expanding device	29 (20.7)	14 (26.4)	0.40
CTR	6 (4.3)	3 (5.7)	0.71
Posterior capsule rupture	5 (3.6)	0 (-)	0.33
Anterior vitrectomy	8 (5.7)	0 (-)	0.11

Data is given as mean (±SD) or absolute numbers and proportions, CDE; cumulative dissipated energy, CTR; capsular tension ring, IFIS; intraoperative floppy iris syndrome, IOL; intraocular lens.

**Table 3:** UCVA and BCVA change at seven and 30 days after cataract surgery.

Variable	Age 50–89 Years *n* = 140	Age ≥90 Years *n* = 53	*p*-Value
**UCVA change (LogMAR)**			
Day 7	−0.35 ± 0.69	+0.02 ± 0.66	<0.001
Day 30	−0.56 ± 0.61	−0.20 ± 0.70	<0.001
**BCVA change (LogMAR)**			
Day 7	−0.39 ± 0.65	−0.04 ± 0.73	0.001
Day 30	−0.55 ± 0.62	−0.27 ± 0.64	0.006

Data is given as mean (±SD). BCVA; best-corrected visual acuity, LogMAR; log of the minimum angle of resolution, UCVA; uncorrected visual acuity.

**Table 4:** UCVA and BCVA at seven and 30 days after cataract surgery.

Variable	Age 50–89 Years *n* = 140	Age ≥90 Years *n* = 53	*p*-Value
UCVA gain ≥ 3 lines			
Day 7	60 (42.9)	13 (24.5)	0.019
Day 30	75 (53.6)	14 (26.4)	0.001
BCVA gain ≥ 3 lines			
Day 7	56 (40.0)	16 (30.2)	0.208
Day 30	73 (52.1)	16 (30.2)	0.006

Data is given as absolute numbers and proportions of eyes improved by at least three lines on the Snellen chart. BCVA; best-corrected visual acuity, UCVA; uncorrected visual acuity.

**Table 5:** One-way analysis of covariance (ANCOVA), including pre-and intraoperative covariates.

Variable	F	MS	*p*-Value	F	MS	*p*-Value
	UCVA Gain at 30 Days			BCVA Gain at 30 Days		
Age (years)	2.801	0.891	<0.001	2.682	0.813	<0.001
Brunescent cataract	1.336	0.425	0.250	2.424	0.735	0.122
Use of α-antagonists	0.000	6.4 × 10^−5^	0.989	0.000	5.6 × 10^−5^	0.989
Age (years)	2.621	0.816	<0.001	2.309	0.717	<0.001
Phacoenergy (CDE)	3.555	1.107	0.062	7.248	2.251	0.008
IFIS	2.127	0.662	0.147	0.805	0.250	0.371
Age (≥90 vs. <90 years)	13.431	5.503	<0.001	8.941	3.510	0.003
Brunescent cataract	2.166	0.887	0.143	2.812	1.104	0.095
Use of α-antagonists	0.034	0.014	0.853	0.013	0.005	0.909
Age (≥90 vs. <90 years)	15.249	6.025	<0.001	9.901	3.812	0.002
Phacoenergy (CDE)	6.752	2.668	0.010	8.872	3.416	0.003
IFIS	2.024	0.800	0.157	1.392	0.536	0.240

ANCOVA was used in an attempt to remove the effect of the pre-and intraoperative covariate factors. Patient age at surgery was analyzed either as a linear parameter (years) or as a binary variable (≥90 [very old patients] vs. <90 years [matched cohort]). Either presence of brunescent cataracts and the use of α-antagonists or the level of phacoemulsification energy and the presence of intraoperative floppy iris syndrome (IFIS) were included as covariates. The F-test test showed a significant relationship between the visual gain at 30 days and age, adjusted for differences in covariates (CDE, IFIS, brunescent cataracts, or use of α-antagonists). BCVA; best-corrected visual acuity in LogMAR units, CDE; cumulative dissipated energy, *F*; between-groups variance divided by within-groups variance, IFIS; intraoperative floppy iris syndrome, LogMAR; log of the minimum angle of resolution, MS; mean square, UCVA; uncorrected visual acuity in LogMAR units.

## Data Availability

Study data is available from the corresponding author upon request.

## Supplementary Materials

The following are available online at https://www.mdpi.com/article/10.3390/jcm10204658/s1, Table S1: Review of studies comparing cataract outcome in elderly in young subjects.
