# Supplementary material for: Cataract Surgery in Very Old Patients: A Case-Control Study
Source: J Clin Med. 2021 Oct 11;10(20):4658. doi: 10.3390/jcm10204658 (PMC8537740; doi:10.3390/jcm10204658)
Supplement: Supplementary file 1 [file jcm-10-04658-s001.zip › jcm-1361569-supplementary.pdf]

Supplemental Table S1. Review of studies comparing cataract outcome in elderly in young subjects.

[1]Author	Year	Country	N eyes (subjects)	Age study group	Control	Matched Ocular co-morbidly	Matched Systemic co-morbidly
Bernth-Petersen[1]	1983	Denmark	55	>90	No	No	No
Mönestam[2]	2004	Sweden	34 (34)	>90	<84; 85-90	No	No
Lundström[3]	2000	Sweden	757	>85	No	-	-
Robbie[4]	2006	UK	54	>90	>88 vs. <88<br><96 vs. <96	NM	NM
Berler[5]	2000	USA	88	>88	<88	No	No
Mehmet[6]	2009	Turkey	30	>90	80-89	No	No
Rosen[7]	2009	Israel	82	>90	No	-	-
Tseng[8]	2011	USA	554	>90	90-89	No	No
Mutoh[9]	2012	Japan	31 (21)	>90	<90	-	No
Sparrow[10]	2012	UK	55,567	>90	<60	No	No
Michalska-Malecka[11]	2013	Poland	122	>90	No	-	-
Lai[12]	2014	Hong-Kong	270	>90	No	-	-
Syam[13]	2014	UK	34	>96	No	-	-
Lee [14]	2018	Korea	44	$\geq$90	No	-	-
Toyama	2018	Japan	138	>90	<80	NM	Yes
Theodoropoulou[15]	2019	UK	25,856 (19,166)	>90	No	-	-
Pahor[16]	2020	Slovenia	90	>90	<90	No	No
Sella[17]	2020	Israel	147	$\geq$85	75-84	Yes*	No
*ocular comorbidities were excluded; NM: not mentioned							

1. Bernth-Petersen P, Ehlers N. Cataract extraction in the “nineties.” *Acta Ophthalmol (Copenh)*. 1983;61:392-396. doi:10.1111/j.1755-3768.1983.tb01437.x.

2. Mönestam E, Wachmeister L. Impact of cataract surgery on the visual ability of the very old. *Am J Ophthalmol*. 2004;137:145-155. doi:10.1016/s0002-9394(03)00900-0.

3. Lundström M, Stenevi U, Thorburn W. Cataract surgery in the very elderly. *J Cataract Refract Surg*. 2000;26:408-414. doi:10.1016/s0886-3350(99)00418-6.

4. Robbie SJ, Muhtaseb M, Qureshi K, et al. Intraoperative complications of cataract surgery in the very old. *Br J Ophthalmol*. 2006;90:1516-1518. doi:10.1136/bjo.2006.098764.

5. Berler DK. Intraoperative complications during cataract surgery in the very old. *Trans Am Ophthalmol Soc*. 2000;98:127-130; discussion 130-132.

6. Mehmet B, Abuzer G. Results of Cataract Surgery in the Very Elderly Population. *Journal of Optometry*. 2009;2:138-141. doi:10.3921/joptom.2009.138.

7. Rosen E, Rubowitz A, Assia EI. Visual outcome following cataract extraction in patients aged 90 years and older. *Eye*. 2009;23:1120-1124. doi:10.1038/eye.2008.203.

8. Tseng VL, Greenberg PB, Wu W-C, et al. Cataract Surgery Complications in Nonagenarians. *Ophthalmology*. March 2011:S0161642010012637. doi:10.1016/j.ophtha.2010.11.023.

9. Mutoh T, Isome S, Matsumoto Y, et al. Cataract surgery in patients older than 90 years of age. *Can J Ophthalmol*. 2012;47:140-144. doi:10.1016/j.jcjo.2012.01.009.

10. the UK EPR user group, Sparrow JM, Taylor H, et al. The Cataract National Dataset electronic multi-centre audit of 55 567 operations: risk indicators for monocular visual acuity outcomes. *Eye*. 2012;26:821-826. doi:10.1038/eye.2012.51.

11. Michalska-Małecka K, Nowak M, Gościniewicz P, et al. Results of cataract surgery in the very elderly population. *Clin Interv Aging*. 2013;8:1041-1046. doi:10.2147/CIA.S44834.

12. Lai FHP, Lok JYC, Chow PPC, et al. Clinical Outcomes of Cataract Surgery in Very Elderly Adults. *J Am Geriatr Soc*. 2014;62:165-170. doi:10.1111/jgs.12590.

13. Syam PP, Eleftheriadis H, Casswell AG, et al. Clinical outcome following cataract surgery in very elderly patients. *Eye*. 2004;18:59-62. doi:10.1038/sj.eye.6700521.

14. Lee YG, Han SY, Han JW, et al. Characteristics of Patients Older than 90 Years Diagnosed with Neovascular Age-related Macular Degeneration. *J Korean Ophthalmol Soc*. 2018;59:444. doi:10.3341/jkos.2018.59.5.444.

15. Theodoropoulou S, Grzeda MT, Donachie PHJ, et al. The Royal College of Ophthalmologists' National Ophthalmology Database Study of cataract surgery. Report 5: Clinical outcome and risk factors for posterior capsule rupture and visual acuity loss following cataract surgery in patients aged 90 years and older. *Eye (Lond)*. 2019;33:1161-1170. doi:10.1038/s41433-019-0389-z.

16. Pahor D, Gračner T. Intraoperative Komplikationen während Kataraktoperationen bei Patienten ab 90 Jahren. *Spektrum Augenheilkd*. August 2020. doi:10.1007/s00717-020-00466-2.

17. Sella R, Chou L, Schuster AK, et al. Accuracy of IOL power calculations in the very elderly. *Eye*. 2020;34:1848-1855. doi:10.1038/s41433-019-0752-0.
